# Toxicity evaluation of zinc aluminium levodopa nanocomposite via oral route in repeated dose study

**DOI:** 10.1186/1556-276X-9-261

**Published:** 2014-05-24

**Authors:** Aminu Umar Kura, Pike-See Cheah, Mohd Zobir Hussein, Zurina Hassan, Tengku Ibrahim Tengku Azmi, Nor Fuzina Hussein, Sharida Fakurazi

**Affiliations:** 1 Laboratory of Vaccine and Immunotherapeutic, Institute of Bioscience, Universiti Putra Malaysia, Selangor 43400, Malaysia; 2 Neurobiology and Genetic Group, Genetic Medicine Research Centre, Faculty of Medicine and Health Science, Universiti Putra Malaysia, Selangor 43400, Malaysia; 3 Department of Human Anatomy, Faculty of Medicine and Health Science, Universiti Putra Malaysia, Selangor 43400, Malaysia; 4 Materials Synthesis and Characterization Laboratory, Institute of Advanced Technology, Universiti Putra Malaysia, Selangor 43400, Malaysia; 5 Centre for Drug Research, Univesiti Sains Malaysia, Penang 11800, Malaysia; 6 Veterinary Science Department, Faculty of Veterinary Medicine, Universiti Putra Malaysia, Selangor 43400, Malaysia; 7 Faculty of Veterinary Medicine, Universiti Putra Malaysia, Selangor 43400, Malaysia; 8 Faculty of Medicine and Health Science, Pharmacology Unit, Universiti Putra Malaysia, Selangor 43400, Malaysia

**Keywords:** Levodopa, Oral toxicity, Nanocomposite, LDH

## Abstract

Nanotechnology, through nanomedicine, allowed drugs to be manipulated into nanoscale sizes for delivery to the different parts of the body, at the same time, retaining the valuable pharmacological properties of the drugs. However, efficient drug delivery and excellent release potential of these delivery systems may be hindered by possible untoward side effects. In this study, the sub-acute toxicity of oral zinc aluminium nanocomposite with and without levodopa was assessed using the Organization for Economic Co-operation and Development guidelines. No sign or symptom of toxicity was observed in orally treated rats with the nanocomposite at 5 and 500 mg/kg concentrations. Body weight gain, feeding, water intake, general survival and organosomatic index were not significantly different between control and treatment groups. Aspartate aminotransferase (AST) in 500 mg/kg levodopa nanocomposite (169 ± 30 U/L), 5 mg/kg levodopa nanocomposite (172 ± 49 U/L), and 500 mg/kg layered double hydroxides (LDH) nanocomposite (175 ± 25 U/L) were notably elevated compared to controls (143 ± 05 U/L); but the difference were not significant (*p* > 0.05). However, the differences in aspartate aminotransferase/alanine aminotransferase (AST/ALT) ratio of 500 mg/kg levodopa nanocomposite (0.32 ± 0.12) and 500 mg/kg LDH nanocomposite (0.34 ± 0.12) were statistically significant (*p* < 0.05) compared to the control (0.51 ± 0.07). Histology of the liver, spleen and brain was found to be of similar morphology in both control and experimental groups. The kidneys of 500-mg/kg-treated rats with levodopa nanocomposite and LDH nanocomposite were found to have slight inflammatory changes, notably leukocyte infiltration around the glomeruli. The ultra-structure of the neurons from the substantia nigra of nanocomposite-exposed group was similar to those receiving only normal saline. The observed result has suggested possible liver and renal toxicity in orally administered levodopa intercalated nanocomposite; it is also dose-dependent that needs further assessment.

## Background

Nanodelivery system is a part of nanotechnology that allows for drugs to be manipulated into nanoscale, allowing for the delivery of drugs to the different parts of the body at the same time retaining the valuable pharmacological properties [1]. This phenomenon, called the ‘quantum effects’, allows for delivery of drugs to areas of the body like the brain in the presence of intact blood brain barrier (BBB) [1]. Layered double hydroxides (LDH) are mainly synthesized via co-precipitation or ion exchange methods [1,2]. They are attracting a great deal of interest as effective and efficient nanodelivery system [1,2]. As a drug delivery system, LDH has a unique controllable ion exchange capacity, pH-dependent solubility, and controlled release properties. These are due to the positively charged metal hydroxide sheets and charge-compensating interlayer anions, hydrated with water molecules of LDH nanocomposite [1]. LDH in drug delivery is said to be less toxic than other inorganic nanodelivery systems [2]; it is generally biocompatible, with both *in vitro* and *in vivo* toxicity studies done to show that [2].

Recent trials have demonstrated a discontinuous and intermittent delivery of levodopa to the brain [3]. This results in the non-physiologic and pulsatile stimulation of striatal dopamine receptors responsible for motor complication seen in Parkinson's disease treatment [3]. Therefore, current researches in the symptomatic treatment of Parkinson's disease focused more on the development of an effective system to deliver levodopa to the brain or new drug formulation with controlled release property [3]. Our group has developed a controlled and sustained release nanodelivery system with levodopa as the active agent [4]. The co-precipitation method was used in the synthesis; it resulted into 16% loading of levodopa into the zinc-aluminium layered hydroxide nanocomposite. The LDH synthesized demonstrated a sustained and pH-dependent release with improved thermal stability. The evidence of levodopa intercalation was demonstrated with the help of X-ray diffraction (XRD) and Fourier transform infrared spectroscopy (FTIR) [4]. Loaded levodopa on the nanocomposite was meant to be taken to the brain, thus, polysorbate 80 (Tween-80) coating of the nanocomposite was conducted [5]. Mediating drugs transportation across the BBB was successfully observed via Tween-80 coating on the surface of some nanoparticles [6,7].

The treatment for Parkinson's disease is lifelong, thus, it necessitates the need for sub-chronic to chronic toxicity evaluation of the current treatment modality. However, no study was done in the past to show the toxic effect of LDH nanocomposite intercalated with levodopa. Thus, this study aimed at the potential clinical, biochemical and histological changes that may ensure following oral administration of zinc aluminium levodopa nanocomposite to Sprague-Dawley rats. The changes were observed over 28 days of repeated dosing with different concentrations of the nanodelivery system.

## Methods

### Animals

Sprague-Dawley rats (250 ± 20 g each) were obtained from in-house animal facility. They were maintained in the animal house of the Department of Anatomy, Faculty of Medicine, Universiti Putra Malaysia, under standard conditions of temperature 25°C ± 2°C, relative humidity 70% ± 5% and 12 h light-dark cycle. The animals were fed with standard rat pellets and tap water *ad libitum*. Throughout the experiments, the animals were ethically handled according to the agreed guidelines for the University's Institutional Animal Care and Use Committee (UPM/IACUC/AUP-RO17/2013: Toxicity and bio-distribution studies of layered double hydroxide, iron oxide nano-particle and single wall carbon nano tube containing levodopa in Sprague-Dawley rats).

### Sub-acute oral toxicity test in rats

The animals were kept in plastic cages for 5 days prior to commencement of dosing, to allow for acclimatization to laboratory conditions. Twenty-eight-day repeated oral toxicity study was conducted as per the Organization for Economic Co-operation and Development (OECD) 407 guidelines [8] with slight modifications in terms of doses administered. Forty animals were randomly distributed into five groups, with each group containing eight rats (Table 1): group 1, zinc-aluminium levodopa high dose (ZALH 500 mg/kg); group 2, zinc-aluminium levodopa low dose (ZALL 5 mg/kg); group 3, zinc-aluminium high dose (ZAH 500 mg/kg); group 4, zinc-aluminium low dose (ZAL 5 mg/kg); group 5, vehicle control (normal saline 100 ml/kg body weight).

**Table 1:** Rats arranged into groups

**Group**	**High dose (500 mg/kg)**	**Low dose (5 mg/kg)**	**Normal saline (100 ml/kg) body weight**
Zinc aluminium levodopa (ZAL)	8 rats	8 rats	NIL
Zinc aluminium nanoparticle (ZA)	8 rats	8 rats	NIL
Vehicle control (VC)	NIL	NIL	8 rats

Arrangement of animals into four treatment groups and one control group, each group with eight rats (*n* = 8). NIL, not given any of the nanocomposite.

Rats in the treatment groups received a dose of freshly prepared nanocomposite (100 ml/kg body weight), while rats in the control group received only normal saline daily. Animal's weights were taken at the start of the dosing (day 0) and weekly thereafter. The animals were observed twice daily for any clinical signs of toxicity and possible mortality during the course of treatment. On day 28 of nanocomposite administration, the animals were sacrificed via exsanguination through cardiac puncture following anaesthesia with ketamine and xylazine. The brain, liver, spleen, heart and kidney harvested from the rats were weighted individually then examined macroscopically for any abnormality.

### Coefficients of the brain, liver, spleen, heart and kidney

The coefficients of the brain, liver, spleen, heart and kidney, which is the ratio of these organs to body weight, were calculated after weighing each organ [the ratio of organ (wet weight, mg) to body weight (g)].

### Biochemical parameters in serum

Blood was collected from rats in each group in a plain 15 mL Falcon tube. It was allowed to stand for about 30 min, before centrifuge at 1,500 rpm, at room temperature. The serum obtained was used for the assessment of biochemical parameters.

### Histopathological evaluation

The animals were subjected to trans-cardiac perfusion using 4% paraformaldehyde (PFA). The tissues obtained were processed using the standard procedure and embedded into paraffin blocks, then microsectioned into 5-μm thick and placed onto glass slides. Haematoxylin-eosin (H & E) staining was used on the tissue sections and viewed using optical microscope (FSX-100 Olympus, Olympus Corporation, Shinjiku-ku, Tokyo, Japan).

### Transmission electron microscope analysis

The substantia nigra was dissected from the whole brain perfused and fixed in 2.5% glutaraldehyde in 0.1 M phosphate buffer (pH 7.2) for 24 h at room temperature, and was washed twice in 0.1 M phosphate buffer. Then the tissues were post-fixed at room temperature for 4 h in a solution containing 1% osmium tetroxide, 0.8% potassium ferricyanide, 5 mM calcium chloride and 0.1 M cacodylate buffer pH 7.2. The tissues were dehydrated in gradient series of ethanol (20% to 100%) and acetone before embedment in epoxy resin at room temperature. The sections for viewing were made into ultra-thin slices using an ultra-microtome, and they were collected on copper grids and stained with uranyl acetate and lead citrate. The sections were viewed with a Hitachi H-600 transition electron microscope (Chiyoda, Tokyo, Japan) (TEM).

### Statistical analysis

The mean values and the standard deviations (SD) of each group were calculated. One way analysis of variance (ANOVA) statistical test was used to compare the groups, and *post hoc* tests were used where there is significant difference to compare between and within groups.

## Results and discussion

### Animal grouping

Rats were arranged into four treatment and one control group at the commencement of the study as shown in Table 1.

### Morbidity and mortality

Morbidity, mortality and gross pathology results of sub-acute toxicity study in rats after repeated oral doses were presented in this study.

### Weight changes during the study

**Figure 1:**
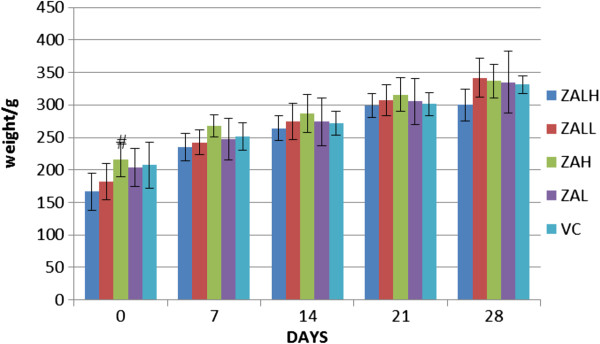
**Average weight gain with time.** The bar chart showed average weight of rats per group at days 0, 7, 14 and 21 of sub-acute toxicity study. There is an obvious increase in the animal's weight; it is shown to be continuous in the four treatment groups as well as the vehicle control. Zinc-aluminium levodopa nanocomposite high dose (ZALH 500 mg/kg), zinc-aluminium levodopa nanocomposite low dose (ZALL 5 mg/kg), zinc-aluminium nanocomposite high dose (ZAH 500 mg/kg), zinc-aluminium nanocomposite low dose (ZAL 5 mg/kg), vehicle control (VC normal saline 100 ml/kg body weight). There is statistically significant difference (#) between day 0 and all other days in all the groups (*p* < 0.05). One-way ANOVA was used, and data are expressed as means ± SD.

The animals treated with zinc-aluminium layered hydroxide nanocomposite intercalated and unintercalated with levodopa over 28 days showed no mortality. The food and water intake in both control and treatment groups were unaffected during the study period. No signs of toxicity, such as vomiting, diarrhoea, paralysis, convulsion, restless, irritation, bleeding and breathing difficulties were observed in any of the groups (Table 2). During the course of experiment, rats treated with high and low doses of nanocomposite showed a sustained weight gain similar to their counterpart in the vehicle control group. The weight gain was shown to be continuous over the study period; statistically, the difference in weight gain between day 0 and all other days in all the groups is significant (*p* < 0.05) (Figure 1). However, body weight changes between weeks were found to be statistically significant (*p* < 0.05), meaning the weight gain in all group from day zero (0) is statistically significant compared to weight in the subsequent weeks. The coefficient of the brain, liver, spleen, heart and kidney was presented in Table 3. It is the ratio of these organs to the whole body taken on the 28th day. There were no significant differences observed in the coefficients of these organs. Thus, 28 days of repeated doses of ZAL and ZA at 5 and 500 mg/kg, via oral route did not show any effect on these organs' weight in relation to the whole body weight. This implies that orally administered ZAL and ZA at 5 or 500 mg/kg respectively do not induce any obvious clinical toxicity or do they resulted in any animal demise.

**Table 2:** Morbidity, mortality and gross pathology results of sub-acute toxicity study in rats after repeated oral doses

**Group**	**Dose (mg/kg) body weight**	**Toxicity sign t/n**	**Mortality d/a**	**Gross pathology l/nl**
ZALH	500	0/8	0/8	0/8
ZALL	5	0/8	0/8	0/8
ZAH	500	0/8	0/8	0/8
ZAL	5	0/8	0/8	0/8
VC	0 (vehicle)	0/8	0/8	0/8

Based on the doses used, no rats showed any clinical toxicity sign, no death was recorded, and no obvious gross pathology seen on the organs observed. Data are expressed as means ± SD, *n* = 8. t/n (toxic/normal), d/a (dead/alive), l/nl (lesion/no lesion). ZALH, zinc aluminium levodopa nanocomposite high dose; ZALL, zinc aluminium levodopa nanocomposite low dose; ZAH, zinc aluminium nanocomposite high dose; ZAL, zinc aluminium nanocomposite low dose; VC, vehicle control.

**Table 3:** Coefficients of the brain, liver, spleen, heart and kidney

**Groups**	**Body weight (g)**	**Brain (mg/g)**	**Liver (mg/g)**	**Heart (mg/g)**	**Spleen (mg/g)**	**Kidney (mg/g)**
ZAL_H_ (*n* = 8)	300 ± 25	5.61 ± 0.93	35.67 ± 1.53	4.00 ± 0.53	1.99 ± 0.37	4.19 ± 0.20
ZAL_L_ (*n* = 8)	342 ± 30	5.76 ± 0.55	36.27 ± 3.35	3.90 ± 0.53	2.08 ± 0.20	4.16 ± 0.22
ZA_H_ (*n* = 8)	337 ± 25	5.62 ± 0.31	30.14 ± 3.54	3.91 ± 0 .43	2.32 ± 0.26	3.98 ± 0. 23
ZA_L_ (*n* = 8)	335 ± 47	5.22 ± 0.68	31.83 ± 4.12	4.50 ± 0.44	2.29 ± 0.19	3.93 ± 0.45
VC (*n* = 8)	332 ± 14	5.31 ± 0.70	28.25 ± 2.71	3.86 ± 0 .35	1.88 ± 0.19	3.59 ± 0.39

Mean coefficient of brain, liver, spleen, heart and kidney of all the groups. The coefficients of organs from the four treated groups were almost similar to those of the control. Statistical test used to compare the means of each group against the control group was done using one-way ANOVA; it shows no significant difference with *p* > 0.05. Zinc-aluminium levodopa nanocomposite high dose (ZALH 500 mg/kg), zinc-aluminium levodopa nanocomposite low dose (ZALL 5 mg/kg), zinc-aluminium nanocomposite high dose (ZAH 500 mg/kg), zinc-aluminium nanocomposite low dose (ZAL 5 mg/kg), vehicle control (VC normal saline 100 ml/kg body weight).

Repeated doses or sub-acute toxicity study is aiming at evaluating target organ toxicity relative to cumulative exposure [9]. These kinds of studies are to be conducted at any point from initial discovery through to late-stage development of drugs and other substance including nanoparticle before clinical trial and human exposure [9]. These studies are conducted to detect potential hazards and assess risk in drug discovery. Aluminium and zinc are the two metals used in the synthesis of this delivery system. Zinc is considered a trace element with multiple beneficial effects especially in the immune system, phagocytosis, intracellular killing and cytokine production by the immune cells [10]. It may also act as an excellent antioxidant, with membrane stabilization ability, preventing free-radical-induced cellular injury [10]. However, multiple administrations of some supposedly valuable compounds at lower concentration may reveal their probability in generating cumulative and latent physiological, biochemical and/or pathological changes [11]. These changes may not be obvious with single toxic or high-dose exposure [11]. Thus, there is the need for in-depth toxicity assessment of this nanocarrier system. Here, it was done using two different concentrations (5 and 500 mg/kg) of the two nanocomposites (ZAL and ZA). The result shown here agreed to a related sub-acute toxicity study results [12] where four different doses of four different sizes of magnesium aluminium layered hydroxide nanocomposite given to mice via intra-peritoneal route for 20 days cause neither mortality nor significant body weight change [12].

Gold nanocomposite (GNP) is another example of inorganic nanodelivery systems that are receiving a lot of attention in nanomedicine [13]. Interestingly, oral administration of GNP to rats produced no marked treatment-related toxicity [14], similar to what was observed here. The nanocomposite was shown to have LD_50_ value greater than 2,000 mg/kg body weight [14]. Generally, data on the acute, sub-acute and chronic toxicity of nanoparticles used in nanomedicine has begun, but they are still at preliminary level and patchy [13].

### Biochemical parameters in serum

**Figure 2:**
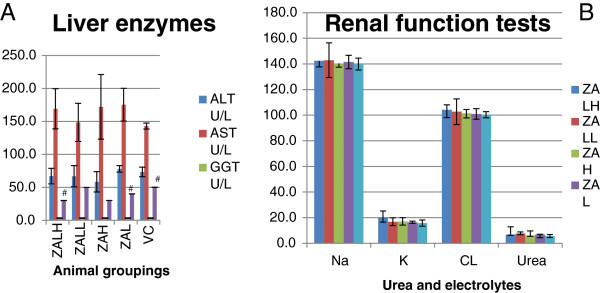
**Effect of ZAL and ZA on biochemical parameters of rats after oral treatment.** Effect of ZAL and ZA on biochemical parameters of rats after oral treatment for twenty eight days using 5 mg/kg and 500 mg/kg doses. **(A)** Liver enzymes. **(B)** Renal function tests. All data are expressed as means ± SD and were compared using one-way ANOVA (*n* = 5). Differences with *p* < 0.05 are considered statistically significant. From the table, AST in ZALH, ZAH and ZAL was notably elevated compared to VC, but the difference were not significant (*p* > 0.05). However, the differences in AST/ALT ratio of ZALH (#) and ZAH (#) were statistically significant compare to VC (#) group. Other parameters measured were found to have no statistical significant difference compared to the control group (*p* > 0.05). ALT (alanine aminotransferase), AST (aspartate aminotransferase), CK (creatine kinase), Creat (Creatinine), GGT (Gamma-glutamyltransferase), Na (sodium), K (potassium), Cl (chloride).

Biochemical parameters from serum were measured to check for any liver and or kidney damage, which may be indicative of injury following repeated doses of the nanodelivery systems. An enzyme of liver mitochondrial and cytosol, aspartate aminotransferase (AST) in ZALH, ZAH and ZAL groups was shown to be elevated compared to VC group, but the difference was not significant (*p* > 0.05) (Figure 2A). However, the differences in aspartate aminotransferase/alanine aminotransferase (AST/ALT) ratio of ZALH and ZAH were statistically significant compare to VC group (*p* < 0.05). Other biochemical parameters measured from the serum of the treated groups were found to have no statistical significant difference compared to the control group (*p* > 0.05).

These parameters were measured in other to assess the effect of the nanodelivery system on the liver, kidney and heart (Figure 2A,B). Elevation of liver enzymes such as ALT, GGT and AST is a part of classical liver cell injury in drugs or of other diseases [15]. Some of these enzymes are not specific to liver cells, as such they are also elevated in other disease conditions or due to injury to the kidney and/or muscle cells [16,17]. The presence of ALT mainly in the cytosol of the liver and its low concentrations elsewhere make it relatively a more specific indicator of liver inflammation than the AST [15]. However, in this study AST elevation was followed by a significant alteration in AST/ALT ratio (Figure 2A). This may indicate a hepatotoxic effect of ZAL and ZA at higher doses via oral route in repeated administration. Previously, an inorganic silver nanoparticle at 125 mg/kg had induced some liver toxicity after oral administration to Sprague-Dawley rats [18]. An inverse dose-related hepatotoxicity was also reported in the past from zinc oxide nanoparticle exposure to mice [19]. This is contrary to the dose-related hepatic injury observed here, although the same administration route was used [19]. The aggregation of these nanoparticles in the liver tissue and subsequent decrease antioxidant functioning system through free radical generation were suggested to be a mechanism in hepatic injury by some nanoparticles [20]. Elevation of enzyme gamma-glutamyl transpeptidase points more towards obstruction to the biliary system. However, in this study the level of GGT was found to be not significantly different between the treated and control groups.

The assessment of renal function becomes imperative and very vital due to the role that kidneys play in drug metabolites and excretion from the body [21,22]. Both zinc and aluminium were incriminated in renal pathology, especially after prolonged usage at higher doses especially in kidney failure patients [23]. Thus, urea, electrolyte and creatinine levels were analysed after the 28-day oral dosing of the rats. They were compared with the control group to see changes. Except for potassium (K) level in the high-dose ZAL nanocomposite group that was slightly elevated, all other electrolytes and urea are within the same range with control group (Figure 2B). Using the 95% confidence interval (*p* < 0.05), none of the parameters measured were found to be significantly different compared to the control group (*p* > 0.05). Creatinine and urea are the by-products of creatine and protein metabolism, respectively. In addition, they are almost completely filtered and excreted out of the body by a normal functioning kidney [24]. Increasing serum concentrations of either or both may correspond with a worsening of the glomerular filtration rate or their increase production in excess of renal ability to handle them [25]. The body homeostasis with regard to Na^+^, K^+^ and Cl^-^ as well as other vital elements in the body can also be useful in evaluating kidney function, because a derange and dysfunctional renal system will alter the electrolytes balance [25]. However, none of the renal functional parameters were significantly altered after oral ingestion of ZAL and ZA.

### Histopathological evaluation

**Figure 3:**
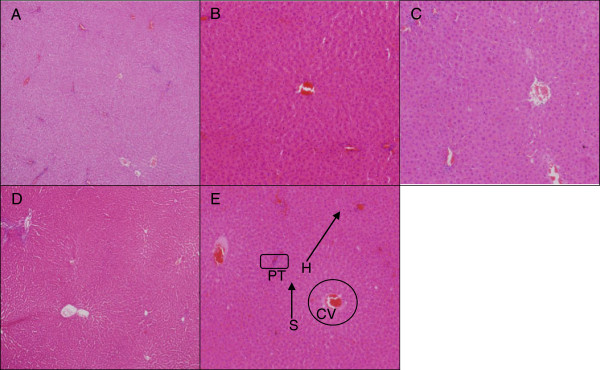
**Microscopic appearance of the liver stained with H & E.** Normal architecture of liver tissue after stained with H & E. The hepatocytes are well delineated with centrally located nucleus, seen in control and the four treated groups. This hepatic histology was taken at ×10 magnification from the rats 4 weeks post treatment with ZALH **(A)**, ZALL **(B)**, ZAH **(C)**, ZAL **(D)** and vehicle control **(E)**. The doses were given via oral route repeatedly over the 28 days study period. Portal triad (PT), central vein (CV), hepatocytes (H) and sinusoid (S). The hepatic lobular array is shown to be well maintained with central vein at the centre surrounded by many portal triads.

Liver histology of the control group showed well-preserved hepatocytes morphology; a well-maintained lobular array with central vein, radiating sinusoids and portal triads were all clearly observed (Figure 3E). The same findings were demonstrated in the treated group from all doses used (Figure 3A,B,C,D). In the case of mild or early liver insult, transferases and or phosphatase levels would be elevated without any clinical symptoms [26]. This may be followed by jaundice, encephalopathy, coagulopathy and possibly some microscopic changes on histology [26]. Here, only slight liver enzymes' derangement was noted at higher doses of ZAL and ZA, and neither clinical nor microscopic evidences of liver toxicity followed.

**Figure 4:**
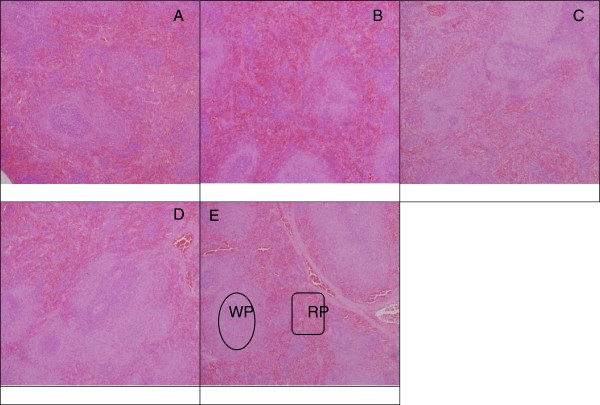
**Microscopic appearance of the spleen stained with H & E.** Normal architecture of splenic tissue on light microscope after stained with H & E. The micrographs were taken at ×10 magnification in rats 4 weeks post treatment with ZALH **(A)**, ZALL **(B)**, ZAH **(C)**, ZAL **(D)** and vehicle control **(E)**. The doses were given via oral route repeatedly over a period of 28 days. White pulp (WP) and red pulp (RP) in experimental groups were shown to be similar in appearance compared to the control.

**Figure 5:**
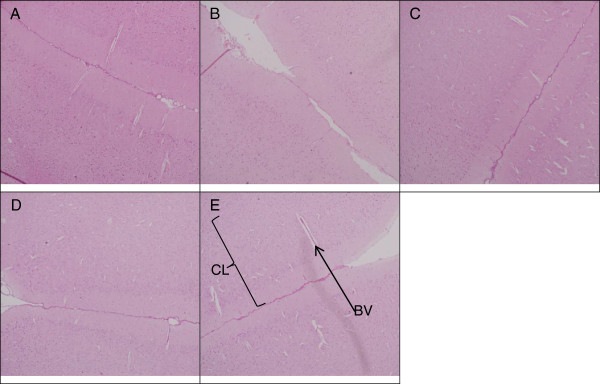
**Microscopic appearance of cerebral cortex stained with H & E.** Histopathology of cerebral cortex (×10) in rats 4 weeks post-exposure to different concentrations of ZALH **(A)**, ZALL **(B)**, ZAH **(C)**, ZAL **(D)** and vehicle control **(E)**. The H & E stained micrographs showing cerebral cortex layers (CL), many neuronal cells and blood vessel (BV) on micrograph **(E)**. Similar structural appearances were noted on all the four treated groups (A to D), thus no changes were seen in the cerebral cortex of the treated animals compared to control.

**Figure 6:**
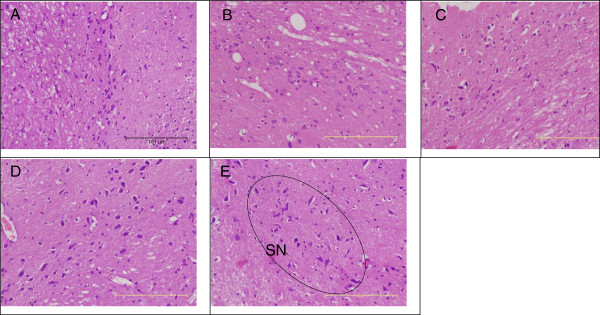
**Microscopic appearance of the substantia nigra stained with H & E.** Microarchitecture of midbrain section (×10) in rats 4 weeks post-exposure to different concentrations of ZALH **(A)**, ZALL **(B)**, ZAH **(C)**, ZAL **(D)** and vehicle control **(E)**. Substantia nigra (SN), with abundant of dopaminergic neurons well outline from the brain of the control rats **(E)**. The brain of all the four treated groups of animals also displayed similar features after H & E stain and viewed at ×10 magnification. No changes were seen in the treated group that could be attributed to the effect of nanocomposite exposure.

The histology of the spleen and brain was modestly similar in the control and experimental groups (Figures 4, 5, and 6). No remarkable changes were seen in the treated group that can be associated to nanodelivery systems ingestion. Two parts of the brain namely the cerebral cortex and the substantia nigra were stained and viewed, this is because of their importance in Parkinson's disease and treatment [27].

**Figure 7:**
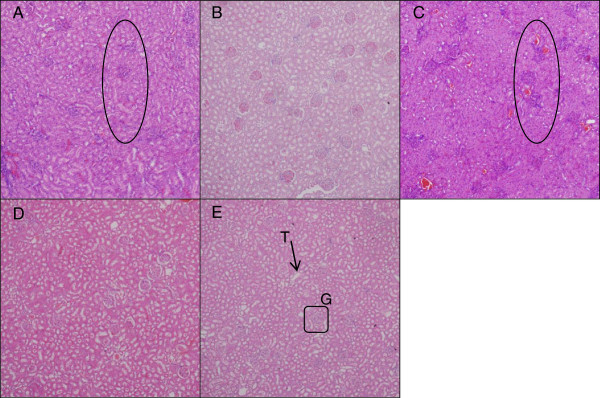
**Microscopic appearance of the kidney stained with H & E.** Microarchitecture of kidney tissues stained with H & E and viewed at ×10 magnification in rats 4 weeks post-exposure to different concentrations of ZALH **(A)**, ZALL **(B)**, ZAH **(C)**, ZAL **(D)** and vehicle control **(E)**. G, glomerular; T, tubule. Micrographs (A) and (C) (encircled areas) show some leukocyte infiltrations which are eosinophilic glomerular due to inflammation likely caused by high dose of the nanocomposite delivery system. The two areas from (A) and (C) were viewed under higher magnification and they are presented in Figure 7.

**Figure 8:**
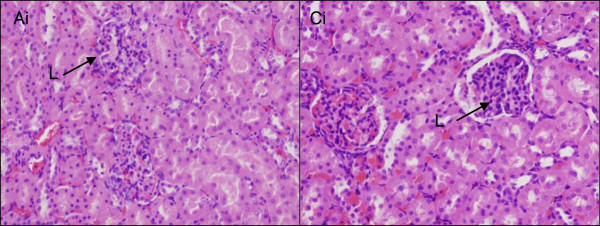
**Microscopic appearance of the kidney stained with H & E.** Histopathology of the kidneys tissue at ×40 magnification in rats 4 weeks post-exposure to different concentrations of ZALH (Ai) and ZAH (Ci). The tissue sections were stained with H & E. Micrographs from the two groups treated with 500 mg/kg of ZAL and ZA, respectively, showing leucocyte infiltration (L) of the glomeruli due to inflammation.

Some inflammatory changes were noticed in kidney sections of ZALH and ZAH groups compared to VC group (Figures 7A, 4B, and 8). Notably, there were some leukocyte infiltrations in both cases. These changes are dose dependent, seen only in the two high-dose-treated rats but not the lower-dose-exposed animals. Drug-induced renal toxicity in the form of inflammation is a common finding [28], some of which are dose related. They can affect the glomerulus, renal tubular cells and/or the surrounding renal interstitium. This finding is also in agreement with the pathological observation in the case of orally administrated zinc oxide nanoparticle to mice [29], where both oral and intra-peritoneal administration of the nanoparticle at different doses demonstrated inflammatory changes in the liver, kidney and lungs [29].

### Transition electron microscopy

**Figure 9:**
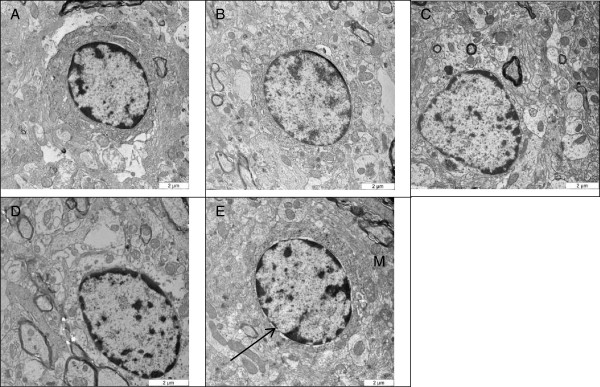
**Micrograms showing neuronal nucleus from the substantia nigra.** TEM ultra-structural micrographs of the rat substantia nigra (*n* = 3) showing the nucleus of a neuron after treatment with **(A)** ZALH, **(B)** ZALL, **(C)** ZAH, **(D)** ZAL and **(E)** VC. Arrow pointing to the intact round-shaped nuclei with a densely peripheral nuclear chromatin condensation (opaque nuclei membrane) and mitochondria (M), with well-outlined cristae and intact opaque membrane in the control group. Similar nucleic and mitochondrial structure and shape were found in the entire treated groups at ×10,000 magnification.

The TEM analysis of the neuronal cells from substantia nigra demonstrated an intact neuron with well-defined nucleus that has a well-delineated peripheral nuclear condensation, which is densely opaque (Figure 9). The shapes were found to be round to ovoid with abundant other cellular organelles notably the mitochondria maintaining its cristae and opaque membrane. In brief, ultra-structural manifestations of the neurons from the substantia nigra were comparatively the same in the control and the nanocomposite-treated groups (Figure 9).

Some nanodelivery-based drug delivery systems were understood to induce oxidative stress characterized by reactive oxygen species (ROS) generation and depletion of antioxidant like glutathione (GSH) usually through free radical generation [1]. However, free radicals were incorporated in the pathophysiology of Parkinson's disease [30]. They were found to cause injury to neuronal cells through damaging DNA, proteins and lipids of the cell or nuclear membrane. These necessitate the need in looking at the neurones from the substantia nigra for these changes after treatment with different doses of ZAL and ZA. Nevertheless, none of the doses used over 28 days cause any cellular damage as seen with electron microscopy. This finding is in agreement with our previous *in vitro* study (32), where the morphology of a neuronal cell (PC12) was preserved despite treatment with IC_50_ concentration of ZAL and ZA over 72-h period. Thus, treatment of Parkinson's disease with zinc aluminium nanocomposite intercalated with levodopa is not likely to worsen the disease condition in future.

## Conclusions

In this experiment, the potential toxicity of zinc aluminium nanocomposite with and without levodopa (ZAL and ZA) on Sprague-Dawley rats after repeated doses was investigated. Rats treated with low and high doses of nanocomposite showed a sustained weight gain similar to their counterpart in the vehicle control group. AST in ZALH, ZAH and ZAL groups was insignificantly elevated compared to VC (*p* > 0.05). However, the statistically insignificant elevation of AST (liver) enzyme was followed by a significant change in AST/ALT ratio of ZALH and ZAH compared to VC group. The kidney sections from ZALH and ZAH showed some leucocyte infiltrations of the glomeruli. This implies that orally administered ZAL and ZA at 5 mg/kg or 500 mg/kg do not cause any obvious clinical toxicity or do they resulted in any animal demise. However, more studies are needed to further assess this new delivery system especially its potential in liver and renal toxicity.

## Abbreviations

ALT: alanine aminotransferase; ANOVA: analysis of variance; AST: aspartate aminotransferase; BBB: blood brain barrier; CK: creatine kinase; Cl^-^: chloride; CV: central vein; FTIR: Fourier transform infrared spectroscopy; G: glomerular; GGT: gamma-glutamyl transferase; GNP: gold nanocomposite; H: hepatocytes; H & E: haematoxylin-eosin; IACUC: Institutional Animal Care and Use Committee; K^+^: potassium; LD_50_: lethal dose; LDH: layered double hydroxide; Na^+^: sodium; OECD: Organization for Economic Co-operation and Development; PFA: paraformaldehyde; PT: portal triad; RP: red pulp; S: sinusoid; SD: standard deviation; T: tubule; TEM: transmission electron microscope; Tween-80: polysorbate 80; XRD: X-ray diffraction; ZALH: zinc-aluminium levodopa high dose; ZALL: zinc-aluminium levodopa low dose; ZAH: zinc-aluminium nanocomposite high dose; ZAL: zinc-aluminium nanocomposite low dose.

## Competing interests

The authors declare that they have no competing interest.

## Authors’ contributions

AUK performed the experiments, data gathering and the initial write-up, CPS, SF, NFH, ZH and TITA were involved result analysis, drafting the manuscript, intellectual revision and gave approval for the final manuscript.
